# Identification of Cold Spots Using Non-Destructive Hyperspectral Imaging Technology in Model Food Processed by Coaxially Induced Microwave Pasteurization and Sterilization

**DOI:** 10.3390/foods9060837

**Published:** 2020-06-26

**Authors:** Aswathi Soni, Mahmoud Al-Sarayreh, Marlon M. Reis, Jeremy Smith, Kris Tong, Gale Brightwell

**Affiliations:** 1 AgResearch, Palmerston North 4442, New Zealand; Aswathi.Soni@agresearch.co.nz (A.S.); Mahmoud.Al-Sarayreh@agresearch.co.nz (M.A.-S.); Marlon.M.Reis@agresearch.co.nz (M.M.R.); 2 School of Food & Advanced Technology, Massey University, Palmerston North 4410, New Zealand; J.R.Smith@massey.ac.nz (J.S.); K.Tong@massey.ac.nz (K.T.); 3 New Zealand Food Safety Science Research Centre, Palmerston North 4442, New Zealand

**Keywords:** hyperspectral imaging, cold spots, microwave, sterilization, Maillard reaction

## Abstract

The model food in this study known as mashed potato consisted of ribose (1.0%) and lysine (0.5%) to induce browning via Maillard reaction products. Mashed potato was processed by Coaxially Induced Microwave Pasteurization and Sterilization (CiMPAS) regime to generate an F0 of 6–8 min and analysis of the post-processed food was done in two ways, which included by measuring the color changes and using hyperspectral data acquisition. For visualizing the spectra of each tray in comparison with the control sample (raw mashed-potato), the mean spectrum (i.e., mean of region of interest) of each tray, as well as the control sample, was extracted and then fed to the fitted principal component analysis model and the results coincided with those post hoc analysis of the average reflectance values. Despite the presence of a visual difference in browning, the Lightness (L) values were not significantly (*p* < 0.05) different to detect a cold spot among a range of 12 processed samples. At the same time, hyperspectral imaging could identify the colder trays among the 12 samples from one batch of microwave sterilization.

## 1. Introduction

Food processing technologies and sterilization regimes work best if they can provide uniform heating across the food being processed. This would not only ensure consistency in delivering quality parameters but would also make sure that each spot gets equal inactivation of any microbial contaminants present to confirm the requisites of food safety. While conventional sterilization involves heating food in cans or retort packages for a set period of time (121 °C at the coldest spot for more than 5 min to ensure an F0 leading to 12 D (12 log cycle) reduction of *Clostridium botulinum* spores) under pressure, they have been reported to have effects on the heat sensitive components of foods [1,2,3]. Microwave pasteurization and sterilization is an emerging thermal technology that combines preheating with hot water and microwave energy (915 MHz) to achieve sterilization in a shorter time as compared to the conventional technologies [4,5]. This technology was initially developed by Washington State University and 915 Labs with the funding provided by the US government and specific food companies [6]. The apparatus used in this study and referred henceforth as the Coaxially induced microwave-pasteurization and sterilization (CiMPAS) system was manufactured by Meyer Burger Germany GmbH (Hohenstein-Ernstthal, Germany) and the industrial microwave parts were manufactured by MUEGGE GmbH (Reichelsheim, Germany). The regime involves a pre-heat cycle that is employed to bring each sample into a uniform temperature before processing, which is followed by bringing the temperature of the treatment chamber to 121 °C while holding for a pre-decided time depending on the F0 targeted. This is followed by a cooling cycle as the last part. F0 is the approximate time of exposure of the sample at 121.1 °C and this can be calculated using the actual exposure time at a variable temperature, which is calculated for an ideal microorganism with a temperature coefficient of destruction equal to 10 °C [7]. A well-reported challenge with microwave processing of food is non-uniform heating [6,8,9]. This drawback has been reduced to a significant extent with sterilization that combines moist heat with the microwave. However, new methods are continuously sought by researchers to identify any non-uniformity in the processed samples. For example, this includes non-destructive hyperspectral technology, chemical markers, and temperature loggers that could be customized for use inside the food package [10,11,12,13]. Non-uniform heating might lead to effects on quality and appearance along with concerns around food safety while minor variation might or might not be a concern to food safety. It is worth detecting the least heated or cooked regions to ensure that these spots receive enough heat to meet commercial sterilization requirements [13]. There are many ways to understand non-uniform heating depending on the food products. This includes the measurement of color change induced by thermal treatment, monitoring the concentration of heat sensitive products, or measuring the inactivation of heat-resistant bacterial spores in food. However, each of these methods have a few limitations and cannot be complete without another confirmatory assay. For example, time temperature metallic sensors are expensive, might slightly interfere with the microwave heating [12], and, depending on size, might not be able to cover all critical spots. Heat-sensitive chemical markers, such as Maillard products, have been used for a long time to indicate heat profiles after processing [12,14]. However, these might have limitations above 100 °C to depict any change in color using colorimetric assays due to limited sensitivity. Chemical marker M2 (4-Hydroxy-5-methyl-3(2H)-fura-none) is one of the products of Maillard reaction among the three products that have been identified as chemical markers namely 2,3-Dihydro-3,5-dihydroxy-6-methyl-(4H)-pyran-4-one(referred to as M-1) and 5-Hydroxymethylfurfural (M-3) [15]. Kinetics of M2 has been validated and reported using the mashed potato food model to identify cold and hot spots as a result of non-uniform heating (if any) after Microwave-assisted thermal sterilization [15,16]. This method with a slight modification in the composition of the food model has been used in this study to identify cold spots generated after coaxially-induced microwave pasteurization and sterilization.

The principle of spectroscopy in the visible and near-infrared (Vis-NIR) spectral region is the interaction of electromagnetic radiation with the sample, involving light absorption. Regular reflectance (specular) where the light incident angle with the sample surface is equal to the angle at which it is reflected means little or no interaction with the samples. External diffuse reflectance captures information about the surface of the sample. In addition, light scattering is due to the interaction of light with the sample [17,18]. The detection of the outgoing photons (as a result of scattering inside of the sample) enables us to identify absorbing/scattering as well as the amount reflected (specular and external diffuse). The balance of outgoing photons compared to incident photons is commonly used as a measure of how much was lost by absorption and scattering as well as the amount reflected. These processes are wavelength-dependent, which makes the use of the entire Vis-NIR spectrum a rich source of information about the chemical and structural characteristics of samples. The combination of Vis-NIRS with imaging techniques (hyperspectral imaging—HSI) enables scanning a region of interest (ROI) with a Vis-NIR spectrum being acquired per pixel in that region [17,18].

HSI has the potential to be used to understand the properties of different food products and non-uniform cooking that is no exception [19]. It offers an excellent option as it is a non-invasive technology. This has previously been used to identify contaminants in fruits [20] and damages due to handling in vegetables [21] contamination in poultry carcasses [22] and red-meat quality and safety grading [17,18]. Recently, new HSI sensors, called snapshot HSI sensors, have been introduced with advantages of low-cost systems, high-speed data collection (ability to work at the standard video rate), and completely portable systems. However, these sensors provide limited spectral features on a short-range of wavelengths. Snapshot HSI systems showed success in many tasks in food processing research such as in meat quality and safety [17,23], and fruit and vegetable classification [24].

In the current study, hyperspectral imaging has been used to identify cold spots in CiMPAS-treated mashed potato and directly compared to the results of color changes due to a Maillard reaction after microwave-induced sterilization. This study is also an attempt to confirm the consistency of this method across three different processing replicates.

## 2. Materials and Methods

### 2.1. Mashed Potato Model Food Preparation

The mashed potato was prepared, as described by Bornhorst et al., [16] by replacing gellan gum with agar and eliminating the addition of calcium chloride. In short, for every 1000 g of mashed potato, 20 g of agar (Roagar, New Zealand) was added to 830 g of boiling water and mixed using a cake mixer at medium speed for 2 min. Then 150 g of mashed potato flakes were added while mixing to avoid the formation of lumps. The mix was cooled to 60 °C, which was followed by the addition of D-ribose (10 g) and lysine (5 g). This was then followed by mixing for another 2 min before being loaded into each of the 12 trays until a weight of 250 g was reached. Trays were then placed in BNB1 pouches (Cryovac, Hamilton, New Zealand) and sealed (230 mBar) in a Multivac C200 vacuum sealer (Multivac NZ Ltd, Auckland, New Zealand) before loading on to the carrier tray in preparation for processing.

### 2.2. CiMPAS Treatment

To understand the possibility of detecting a difference in heat achieved at various locations, the apparatus for coaxially-induced microwave pasteurization and sterilization (CiMPAS) technique was used. CiMPAS goes through the three steps in the sterilization protocol, which include preheating, hot water immersion, and microwaving followed by cooling. The CiMPAS tool was initially preheated by running a heating program. Then the hot water vessel was stabilized at 130 °C and the warm water vessel was stabilized at 30 °C, respectively, with a +/−0.5 °C tolerance on both. Packaged products were then loaded into a carrier tray, which was placed in the tool and processed using the sterilization regime. As the first step, the vessel was flooded with warm water at 30 °C for preheating the food products for 20–30 min. Following the preheating step, hot water was flushed into the vessel, microwave power was switched on at 12 kW, and the carrier tray was passed through antennae for a set period. This was followed by cooling water (30 °C) being flushed into the vessel to cool the product. Processed trays were removed from the carrier tray and placed into the chiller (4 °C) overnight before analysis. Samples were collected from three processing runs conducted on three different days separately for colorimetry and HSI analysis. Controls were exposed to similar storage conditions except CiMPAS processing.

### 2.3. Hyperspectral Imaging System

**Figure 1:**
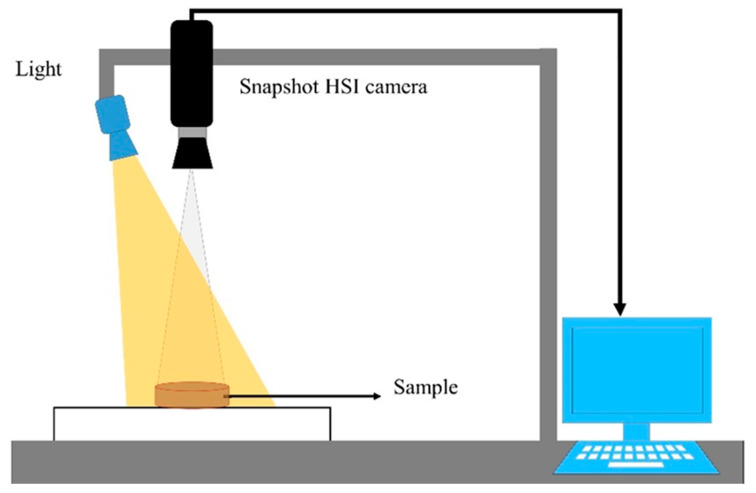
Demonstration of the implemented snapshot hyperspectral imaging (HSI) system.

A low-cost snapshot hyperspectral imaging system in the reflectance sensing mode was applied for image collection. The snapshot HSI system, as shown in Figure 1, consisted of a sample stage and a snapshot hyperspectral camera (MQ022HG-IM-SM4 × 4-VIS [3,12]), which acquires data of 16 wavelengths in the spectral range of 466–639 nm, an illumination unit with a 70-watt quartz-tungsten-halogen (ASD Illuminator unit) (Malvern Panalytical Ltd, UK), and computer running image acquisition software (HS Imager) (Headwall Photonics, Massachusetts, USA). The distance between the camera and the sample stage was set to 60 cm. This value was empirically assessed and then synchronized with the camera by adjusting the exposure time and the frame rate of the camera to 5.1 ms and 7 images/second, respectively, which resulted in images with a spatial resolution of 0.37 × 0.37 mm/pixel.

The exposure time of the camera was adjusted to prevent the saturation of the sensor. The threshold to prevent the saturation is ‘511’ (i.e., any intensity over 511 is defined as saturated). The value of ‘511’ was estimated by the sensor’s manufacture based on size of each pixel in the sensor. The exposure time was adjusted based on the measurement of white tile used as a white reference, which is explained below.

Those configurations were used for collecting the raw HSI images *R*_0 followed by calibration of the reflectance value as follows:

(1) $$R=\frac{\left(R_{\_0}-D\right)}{\left(W-D\right)}\times C$$

where *R* is a calibrated image, *R*_0 is the raw image irradiance, *D* is dark reference data of the sensor, and W is the white reference data of the light source. The dark reference is a hyperspectral image collected when the camera was closed with its cap. Similarly, the white reference is an image of a standard white tile. These reference measurements are used to reduce the impact of experimental variation in the setup and lighting source. The ratio $\frac{\left(R{\_}_{0}-D\right)}{\left(W-D\right)}$ defines a scale between ”zero” corresponding to a dark reference and “one” corresponding to a white reference. The scaler *C* (equal to 511) is used to retrieve the original scale of the HSI sensor as “511” corresponds to a maximum value for non-saturated pixels.

A collection of HSI images, by the implemented system, were acquired for each tray individually in each processing replicate, which resulted in 36 HSI images including 12 images for each replicate. These images were then processed as per Equation (1) for obtaining the reflectance values.

### 2.4. Image Segmentation

**Figure 2:**
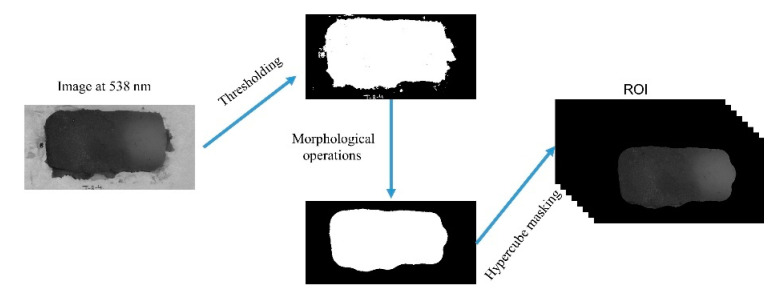
Return on investment (ROI) extraction processes for mashed-potato hyperspectral imaging (HSI) images.

After obtaining the images in reflectance, image segmentation was performed to extract the (ROI). The segmentation process includes several image processing operations including image thresholding using a specific numerical value (reflectance intensity) at a particular wavelength (536 nm) to remove the pixels that belonged to the background and then a set of image morphological operations for removing the noisy pixels and maintaining the shape of the samples. The sequence of these operations included closing, opening, filling holes, erosion, and dilation. The collected HSI images were masked by these segmentation steps for obtaining HSI images with only return on investments (ROIs) for further analysis (Figure 2).

### 2.5. Colorimetric Analysis

**Figure 3:**
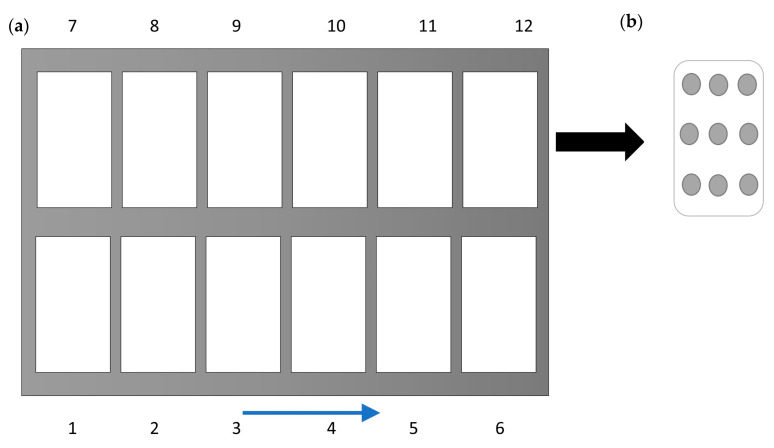
Tray configuration for colorimetric analysis (**a**), where the numbers indicate their position in the processing tray and the arrow indicates the direction toward the waveguide. Division of each tray into nine regions for measuring L values (**b**).

Mashed potato model food samples (in trays) after CiMPAS processing were used for the colorimetry analysis on the three different layers of the product inside. A meat slicer with a uniform height was used to cut the middle slice and nine spots on each layer (top, middle, and bottom) were mapped in each tray for measuring the change in color. The top and bottom layers were then excluded after the preliminary assay as a heat/color pattern on both the top and bottom layer due to being in contact with the immersion water at 12 °C showing uniform browning. The mid-layer, which was more dependent on microwave penetration, was considered to be a better indicator. Each tray was divided into nine different spots as represented by the dots in Figure 3. The change in color of the samples was estimated using L*a*b* (CIELAB) color space using a CR20 colorimeter (Minolta, Osaka, Japan). The aperture of the measurement tool was placed on the samples while taking care to avoid any light penetration through gaps and a cling wrap transparent sheet was used to cover the aperture to avoid liquid penetration into the lens. Calibration was done every time using a white tile before a new sample measured. The lightness (*L values) using *L a B space was recorded as previously reported [16,25]. The coldest spot was determined as the location with the highest (significant at 95% confidence level) L value.

### 2.6. Validation: Effect of Temperature Increase on Browning

**Figure 4:**
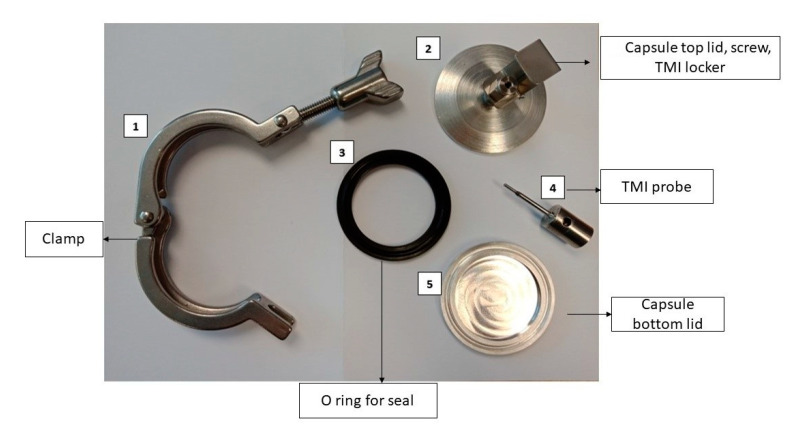
Capsule set up for the oil bath work with the main parts.

For the kinetic study, Digital High-Temperature Oil Bath (Interlab, Wellington, New Zealand) was set at 121 °C. To measure the come-up time, three capsules (Figure 4) with mashed potato and pre-set TMI probes inserted were used.

Once the come-up time was determined, six capsules filled with mashed potato (15 g) were immersed in the oil bath while making sure there was no dripping or leakage from the capsules. Capsules were removed at an interval of 0, 2, 4, 6, 8, and 10 min. This was followed by immediately cooling in an ice slurry to avoid any further reactions due to residual heat. Three experimental replicates were conducted under the same setup. The change in color for each sample was estimated as described in Section 2.5.

### 2.7. Data Analysis

#### 2.7.1. Statistical Analysis of Images

Principal component analysis (PCA) is an established chemometric technique for dimensionality reduction and visualizing HSI data [21]. In the current study, a PCA was used to visualize the variation in the distribution of the heating pattern across the 12 trays, which were processed by one processing run as one replicate. Three processing replicates were considered. By applying PCA to the data, the two-dimensional data was converted into a two-dimensional matrix where each pixel can be converted into one row of reflectance values. This was further used to obtain the Eigenvalues or scores. To obtain a good fit into the PCA model, the average ROI for each image was not suitable as it generated a spectrum for each tray that could not represent the spatial variation. As a solution, super-pixel segmentation approaches have been previously used for converting the image into a set of non-overlapped regions (super-pixels), where pixels in each region share the same spectral and spatial (neighboring) information [26]. In the current study, a simple linear iterative clustering (SLIC) algorithm [26] was selected and adapted for generating the overlapped regions in an HSI image, where the Simple Linear Iterative Clustering (SLIC) algorithm was originally proposed for RGB images. In the SLIC algorithm, super-pixels are generated by clustering pixels based on their spectral similarity and proximity in the 2D plane of the image. The similarity is defined as the Euclidean distance in spectral and spatial domains (spectral response + xy), where the spectral response is the pixel reflectance vector (16 wavelengths) and xy are the coordinates of the pixel in the image plane. The Euclidean distance in a spatial domain is normalized by the maximum distance between any two segments because the distance is not limited and depends on the image size. The algorithm takes two input parameters, which include a desired number of clusters (superpixels) and compactness parameter to control the shape of resulting clusters. In the clustering processes, the centers of clusters were initialized at regular grid intervals in the image plane. Followed by this, in an iterative process, the cluster centers were updated and the pixels belonging to a specific cluster were defined based on the similarity of cluster centers and pixel values (i.e., reflectance vector and x-y coordinates).

**Figure 5:**
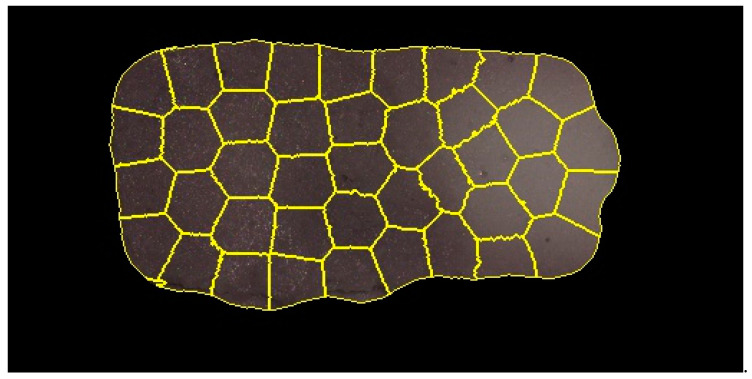
Non-overlapping regions of a mashed-potato HSI image.

In this study, the SLIC algorithm [26] was used for over-segmenting the extracted ROIs into a set of non-overlapping regions (super-pixels) for each HSI image of the middle layer of the mashed-potato (Figure 5) after CiMPAS processing. The desired number super-pixels and the compactness parameter were set into 50 and 0.4, respectively. The values of these parameters were empirically selected to obtain super-pixels that approximately covered only dark or bright pixels in the image. Importantly, the large super-pixels (i.e., low number of super-pixels) could cover regions with mixing of dark or bright pixels in the super-pixel, which could affect the analysis of identifying cold and hot regions in the tray, while small super-pixels (i.e., high number of super-pixels) could increase the impact of noisy pixels in the analysis. The mean spectrum of each segment was extracted and used for fitting the PCA model and the HSI image of replicate 1 was used to fit the PCA model by using 600 spectra (on average of 50 segments in each tray of the 12 trays) for covering both spatial and spectral information of this replicate. The other trays were used for validating the results of the fitted PCA model.

#### 2.7.2. Visualization

The impact of heating was investigated using heating map graphics. For generating these heating maps, the fitted PCA model was used to project spectra corresponding to all pixels from each hyperspectral image onto their PCA space. The color scale for this map was defined based on heat treatment. Thus, the PCA scores corresponding to the control samples are defined as the zero (no heat treatment). As a result, it is possible to define a direction of growth on values of scores regarding the application of heat. For example, if the scores in a principal component for the control samples are higher than the scores of samples in the heat-treated samples, it means the direction of heat application corresponds to a decrease of score values and vice versa. To enhance the visualization of the effect of heat treatment, the scores of heat-treated samples were re-scaled between 0 and 100, using a minimax approach, where zero indicates the coldest regions while 100 indicates the hottest regions. In this case, score values that are closest to values from control samples corresponds to ‘zero’ (lowest amount of heat treatment) and those with score values furthest from values corresponding to control samples are ‘100’ (highest amount of heat treatment).

#### 2.7.3. Statistical Analysis of Colorimetry Results

The significant difference (*p* < 0.05) among the *L values (Lightness values) was estimated using One-way ANOVA and then post hoc analysis at a 95% confidence level to see which values were different from each other. This method was used to understand the average difference in L values across 12 trays per replicate and to understand the difference between nine different regions that were measured per tray. Three processing replicates were used separately, and three technical replicates were averaged for analysis in each processing replicate.

## 3. Results and Discussion

The amount of heat penetration or distribution in food post CiMPAS processing depends on several factors including (but not limited to) dielectric properties of food [27], which could be related to the salt content [28], moisture content [29], and the waveguide distribution inside the treatment chamber [15] along with the temperature of the immersion water and pressure. Browning through a Maillard reaction has been reported to be an indicator of non-uniform heating [11]. However, the drawback remains in the lack of sensitivity using colorimetric analysis, which reduces the limit of detection. To improve the sensitivity, mashed potato samples were simultaneously analyzed by hyperspectral imaging as well as by colorimetry (*L values). For the analysis, each processing replicate was considered as a separate set of work and, for the data analysis, technical replicates were used. This not only removed unclear normalization of the data but also helped to detect the variation in regions across three replicates.

### 3.1. Kinetic Assay to Understand the Effect of Heat on Browning

**Figure 6:**
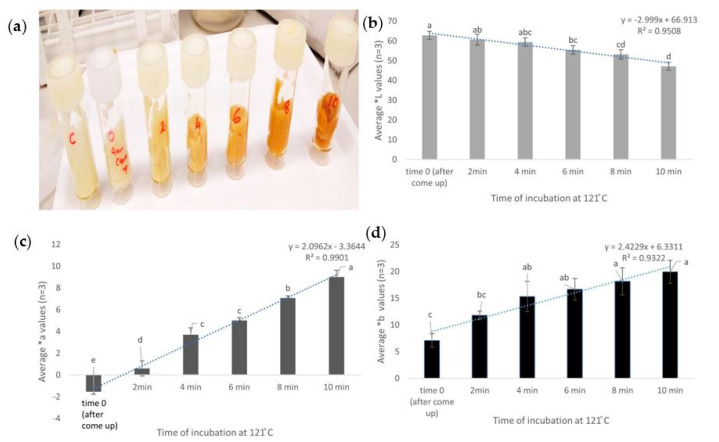
Mashed potato samples with M2 after exposure up to 10 min at 121 °C. The visual change in color (**a**), change in *L values (**b**), change in *a values (**c**), and change in *B values (**d**). The similar letters in each graph indicate no significant difference (*p* < 0.05).

The Maillard reaction has been reported as a reaction in which a reducing sugar (ribose) condenses with a compound possessing a free amino group (amino acid) to give a condensation product, which is an N-substituted glucosamine. N-substituted glucosamine further gets rearranged to form the Amadori rearrangement product (ARP) [30]. This reaction depends on the pH. In neutral and acidic pH, it forms a furfural whereas, in alkaline pH along with many other reactive fission compounds, it forms a furanone. This is again followed by a range of chemical reactions, which can include cyclisation, dehydration, retro-aldolisation, rearrangements, isomerization, and further condensations. In the final stage, they form polymers called meladonins that lead to the formation of a brown color. Chemical marker M-2 (4-Hydroxy-5-methyl-3(2H)-furanone) has been reported as an effective tool to monitor heating patterns of foods in microwave sterilization [15,16]. However, to understand the browning at different layers of the mashed potato gel, a firmer gel was formulated by increasing the concentration of agar (2% of total volume). To verify the pattern of browning as an effect of an increase in time of exposure at a pre-defined temperature of 121 °C, the kinetic assay was used. The come-up time of mashed potato to match the temperature of the surrounding oil was found to be 4 mins, which was estimated by averaging the time taken by three independent mashed potato samples to reach 121 °C (data not shown). There was no significant difference in the *L, *a, or *B values of mashed potato before and after the come-up time. However, the color changed (visually toward browning with an increase in exposure time at 121 °C (Figure 6a). The lightness (*L) values showed a significant (*p* < 0.05) decrease and a linear reduction with time (R2 = 0.95) (Figure 6b). The *a axis represents the green–red component with green in the negative direction and red in the positive direction. The *a values showed a significant (*p* < 0.05) increase from 1.5 at time zero to nine after 10 min (Figure 6c). The b* axis represents the blue-yellow component with blue in the negative direction and yellow in the positive direction. The b values did not show an upward gradient where samples after four, six, eight, and 10 min were not significantly different from each other, which indicated a saturation in values (*p* < 0.05). This indicated that b values would not be an appropriate measure to understand the change in color in mashed potato samples (Figure 6d).

This finding was in agreement with the previous report by Bornhorst et al. [16] when temperatures up to 100 °C were taken into consideration. In the current study, the temperature tested was 121 °C and visual dark browning was seen within 10 min.

Browning increased with exposure at 121 °C (tested up to 14 min, including come-up) and any difference in heat exposure reflected as a change in color (Figure 6). While these findings indicated that heat has a significant effect on browning, the next step involved the analysis of this effect on heat generated by microwave sterilization.

### 3.2. Identification of Cold Spots Post CiMPAS Processing

The CiMPAS processing tray consists of 12 slots for trays where each tray containing 250 mg of mashed potato after vacuum sealing. Each tray was first distributed into three layers: top, bottom, and mid-layer. Each layer was assigned nine spots for measurement of the color change. The nine spots on the mid-layer in each tray were measured and the average *L values (*n* = 9) per tray was used to compare the 12 trays, which could be accommodated in one processing run. Initially, the average L values of nine spots in each tray were compared to the average L values of each of the other 11 trays and control. On the other hand, a comparison by colorimetry to identify browning can give us indicative results for a tray but not for a large sample set. There was no significant difference among the L values of the 12 trays and this was reproducible across three processing replicates (data not shown).

**Figure 7:**
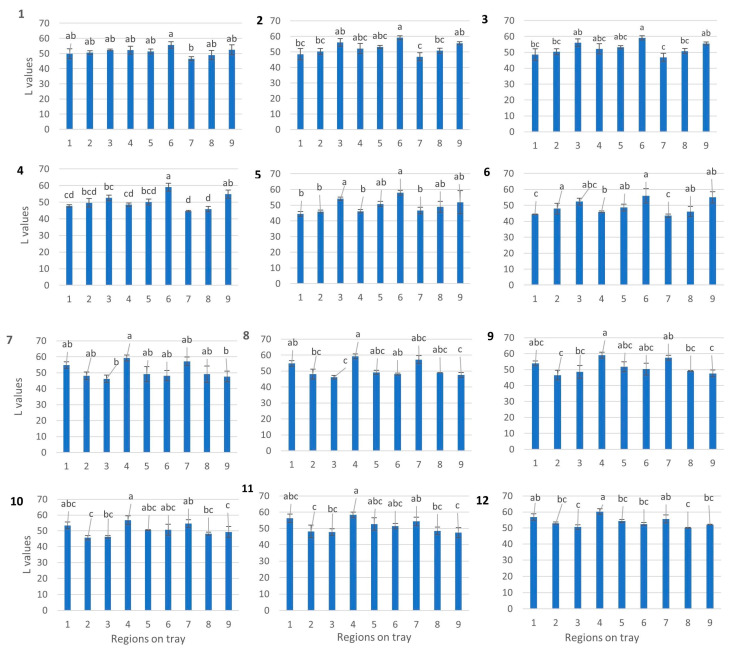
Mid-layer analysis of each tray after processing at 121 °C. Similar letters indicate no significant difference (*p* < 0.05).

However, when the average *L values (*n* = 3 processing replicates) of the nine spots were compared to each other in each tray and hot and colder regions were identified based on significantly (*p* < 0.05) lower and higher *L values, respectively (Figure 7). Though there was a difference in heating patterns, it was not significant enough to understand a colder region among the 12 trays of the processing vessel. However, the colder spots on each tray were identified (Figure 3). These spots spanned both the outer regions of the processing tray where the trays would encounter the surrounding water. Though the water is at 121 °C most of the time, there is a preheating and cooling cycle, which holds the water at 60 and 30 °C and this would explain why the browning indicated a difference on the outer sides. To increase the sensitivity of the assay and to be able to look at the difference when all the 12 trays were being compared, hyperspectral image analysis was used.

### 3.3. Hyperspectral Image Analysis

**Figure 8:**
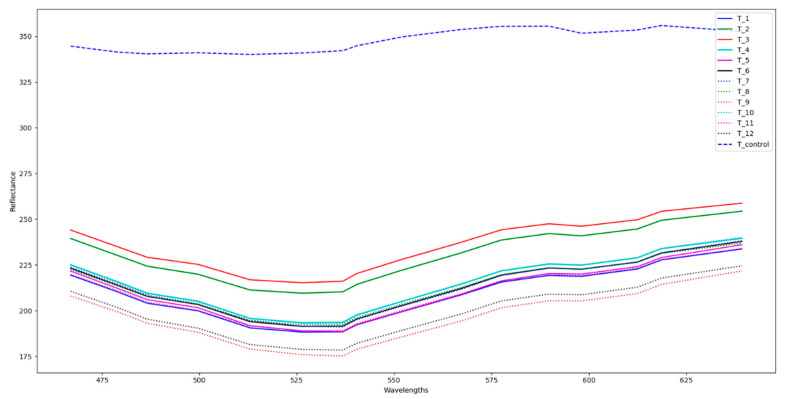
Average spectra for 12 processed trays and one control of mashed potato.

The average spectrum of regions in the tray results from HSI presented in Figure 8, where a clear difference between the control and heat treat samples is observed. Overall, control samples present a higher reflectance.

**Figure 9:**
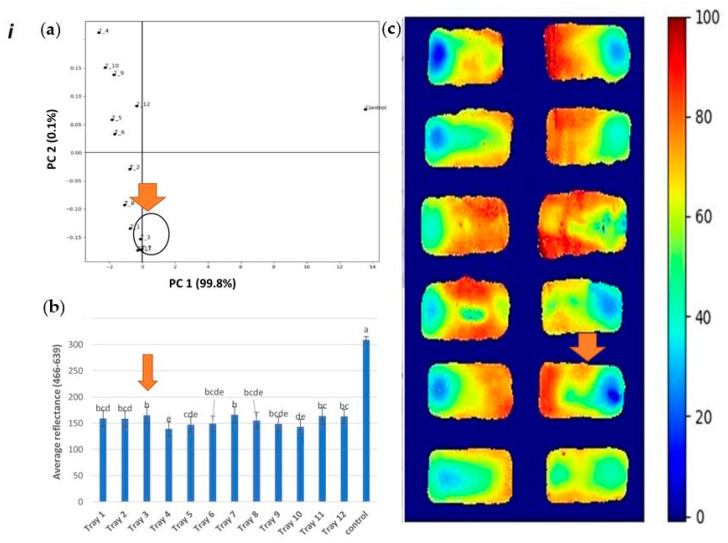

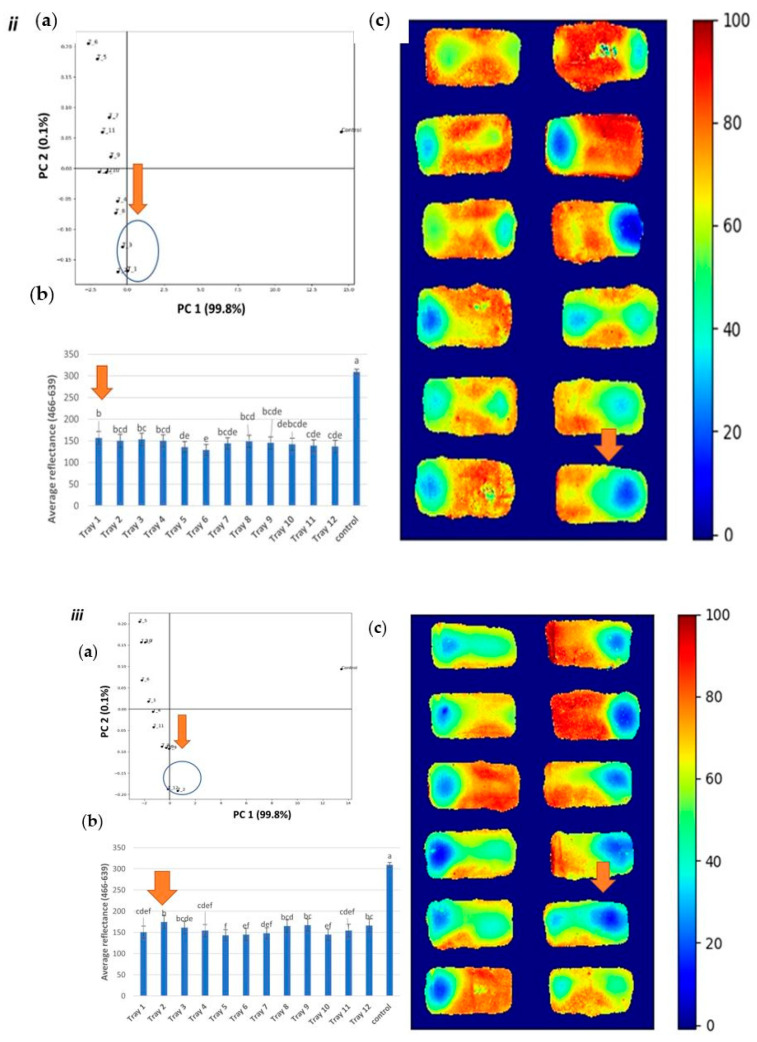
Hyperspectral analysis of processing runs (i, ii, and iii): PCA plot (**a**) average reflectance (**b**) and heat map (**c**) as a comparison of 12 trays (tray number 1 to 12 indicates its location in the processing vessel), where the image c in each figure indicates ‘zero’ (lowest amount of heat treatment) and ‘100’ (highest amount of heat treatment).

The scatter plot of scores from both principal component (PC) 1 and PC 2 are presented in Figure 9, where it is shown that PC 1 captures the difference between control and heat-treated samples. In this plot, the control samples present high positive values and heat-treated samples present negative values. Thus, PC 1 was used to develop the heating maps. In this case, scores in PC 1 are close to zero and correspond to areas least affected by heat-treatment, herein defined as cold, while scores in PC 1 that are furthest from zero correspond to areas more affected by heat-treatment, herein defined as hot. The visual representation that consisted of the hyperspectral data converted to a heat map clearly showed a difference in the heating pattern within each tray as well as in the whole processing tray consisting of 12 trays (Figure 9ic–iiic). The high variance described by the PC 1 (99.8%) suggests high correlation among variables and/or high correlation among samples. This indicates a linear variation in the concentration of pigments associated with the changes in color and that the ratio among these pigments did not vary due to variation in the temperature of different regions in the sample.

Hyperspectral imaging was found to be time-efficient in data acquisition as compared to the colorimetry and concurrently for increasing the limit of detection and ruling out wrong negatives due to a huge variation of the colorimetry results (Figure 9i–iii).

Clearly and in agreement with the above statistical analysis, these plots show that, for all replicates, the cold and hot spots are projected on the same location in the PCA space with the approximately same distance from the control sample. For instance, in the first replicate (Figure 9i), tray 3 was subjected to be the coldest region as per the average reflectance (*p* < 0.05) and PCA analysis. Similarly, in replicate 2 and 3, tray 1 and 2 were subjected as the coldest regions, respectively. These trays were close to each other and near the door in the processing set-up, which also indicates that this would be the colder region that accumulates less heat as compared to the regions away from the door.

The observations support the potential to use a snapshot HSI imaging system for visualizing the heating processes of the processed food like mashed potatoes, especially where colorimetry (*L values) might not be sufficiently sensitive. Prediction of the colder regions through hyperspectral imaging would not only indicate regions that could lead to a significant difference in sensory food attributes but can also be used as a tool to identify critical spots for inoculation of bacterial sterilization indicators, which are required for validation of any thermal process. The results were also in agreement with the previous study by Pan et al. [10], where HSI (400–1050 nm) was investigated as a method to identify non-uniform regions post microwave sterilization and the results were comparable to those obtained using an infrared (IR) thermal imaging technique. However, the food matrix itself can induce noise in the results obtained. Hence, a comparison with control samples of each batch would help rule false results.

## 4. Conclusions

Hyperspectral image analysis was successfully able to increase the limit of detection to identify colder regions in the processing tray with 12 trays of mashed potato model food after CiMPAS processing, while colorimetry could not identify these colder regions. This also confirms the use of spectral modelling as a tool for cold spot detection. The results showed consistency in detection when samples from three independent processing runs were analyzed. Hence, the detection of the worst critical points via non-destructive HSI indicates the potential to identify critical colder spots, which forms an essential part of ensuring the consistency of microwave-induced sterilization. It also indicates a potential for research on modelling a wide range of food that cannot be formulated as a model system or spiked with a heat-sensitive biomarker.
